# Machine Learning-Enabled Smart Industrial Automation Systems Using Internet of Things

**DOI:** 10.3390/s23010324

**Published:** 2022-12-28

**Authors:** Ali M. Al Shahrani, Madani Abdu Alomar, Khaled N. Alqahtani, Mohammed Salem Basingab, Bhisham Sharma, Ali Rizwan

**Affiliations:** 1 Faculty of Computer Studies, Arab Open University, Riyadh 11681, Saudi Arabia; 2 Department of Industrial Engineering, Faculty of Engineering at Rabigh, King Abdulaziz University, Jeddah 21589, Saudi Arabia; 3 Department of Industrial Engineering, College of Engineering, Taibah University, Madina 41411, Saudi Arabia; 4 Department of Industrial Engineering, Faculty of Engineering, King Abdulaziz University, Jeddah 21589, Saudi Arabia; 5 Chitkara University Institute of Engineering and Technology, Chitkara University, Rajpura 140401, Punjab, India

**Keywords:** industrial automation, robotics, Internet of Things (IoT), machine learning, elaborative stepwise stacked artificial neural networks (ESSANN) algorithm, industrial environment, principal component analysis (PCA), least absolute shrinkage and selection operator (LASSO)

## Abstract

Industrial automation uses robotics and software to operate equipment and procedures across industries. Many applications integrate IoT, machine learning, and other technologies to provide smart features that improve the user experience. The use of such technology offers businesses and people tremendous assistance in successfully achieving commercial and noncommercial requirements. Organizations are expected to automate industrial processes owing to the significant risk management and inefficiency of conventional processes. Hence, we developed an elaborative stepwise stacked artificial neural network (ESSANN) algorithm to greatly improve automation industries in controlling and monitoring the industrial environment. Initially, an industrial dataset provided by KLEEMANN Greece was used. The collected data were then preprocessed. Principal component analysis (PCA) was used to extract features, and feature selection was based on least absolute shrinkage and selection operator (LASSO). Subsequently, the ESSANN approach is proposed to improve automation industries. The performance of the proposed algorithm was also examined and compared with that of existing algorithms. The key factors compared with existing technologies are delay, network bandwidth, scalability, computation time, packet loss, operational cost, accuracy, precision, recall, and mean absolute error (MAE). Compared to traditional algorithms for industrial automation, our proposed techniques achieved high results, such as a delay of approximately 52%, network bandwidth accomplished at 97%, scalability attained at 96%, computation time acquired at 59 s, packet loss achieved at a minimum level of approximately 53%, an operational cost of approximately 59%, accuracy of 98%, precision of 98.95%, recall of 95.02%, and MAE of 80%. By analyzing the results, it can be seen that the proposed system was effectively implemented.

## 1. Introduction

**Figure 1:**
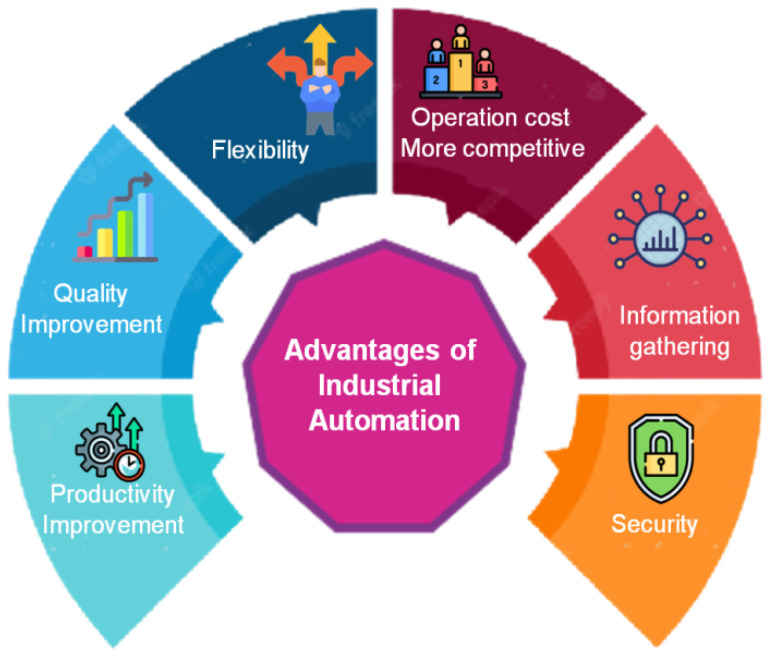
Advantages of industrial automation.

The Industrial Internet of Things (IoT) is a term used to describe a linked system of interrelated detectors, equipment, and other components to enhance automated industrialization. This enabled system surveillance and remote control. The primary function of IoT is to improve the industrial automation process. The IoT concept is utilized to assess, turn on, and manage various machines in the shipping, automotive, textile, agricultural, food, and beverage industries. Using data gathering, data from many sources can be shared and analyzed. The acquired data must be analyzed using smart functions to transmit alerts or triggers to other systems. IoT is used to provide fresh concepts for challenges and improve the effectiveness of the procedures. It focuses on maintaining efficient interfaces and interactions using controllers, robots, and monitors. Concepts from the Industrial Internet of Things were used to improve the effectiveness of the process. There have been reports on digital industrialization and its role in product design. Software and IoT idea modules have been used in contemporary industrial processes [1]. The adoption of IoT across several industrial sectors has increased exponentially over the last few decades. According to Gartner, by 2025, there may be 24 billion IoT gadgets. These gadgets create enormous amounts of data that require effective storage and processing. Increased machine-to-machine (M2M) and direct-to-device (D2D) connections are also a part of this process, which involves data sharing. A strong IoT standards stack that can manage all problems with data transportation and analysis at different stages is required to address this massive data expansion. A structure may be created with the aid of standardized protocols and layers for IoT devices to perform necessary functions. To satisfy customer requests and accomplish conservative business objectives, the automobile industry has aggressively adopted these gadgets [2]. Figure 1 depicts the advantages of industrial automation.

Many types of businesses and industries utilize industrial automation to develop more effective operations using various technologies. Industrial applications are replacing some of the routine tasks that people perform. This frees workers to undertake higher-level activities, while enabling repetitive and precise tasks to be accomplished with fewer mistakes. The modernization of the equipment and systems utilized in sectors such as production and manufacturing is known as industrial automation. The aim was to minimize the number of tasks carried out by people. The use of industrial automation technology by organizations improves security, frees up time, improves product quality, minimizes monitoring, and reduces costs. All these advantages help businesses operate more profitably, productively, and efficiently [3]. Industries have been using a well-defined five-layer automation infrastructure for the past 30 years or more, where smart monitors and I/O (such as sensors) at the lowest point communicate with the conceptual processors using an analog signal Programmable Logic Controller (PLC). Remotely controlled duties are performed using Supervisory Control and Data Acquisition (SCADA) systems. Users using Manufacturing and Execution Systems (MES) can perform difficult activities, including production planning and reliability control. ERP (Enterprise Resource Planning (ERP) systems at the highest level provide managerial monitoring and communicate production data, such as order progress, with other software, such as Customer Relationship Management (CRM) systems. This is the traditional automation infrastructure [4]. 

Industrial automation solutions are evolving into more complicated Cyber-Physical Systems (CPS), owing to Industry 4.0. The integration of systems across horizontal and vertical levels in industrial and disciplinary areas is very important. In addition to high-level structure and regulation at the implementation network level, the construction of industrial CPS necessitates Offline and Online Engineering (OOE) work, which includes designing the device’s mechanical and electrical components, as well as their setup, connectivity, and information. An integrated solution to handle OOE duties using DevOps, a set of contemporary software lifecycle administration techniques, is necessary. The development, testing, and implementation of software services are coordinated through Constant Integration/Constant Deployment (CI/CD) pipelines and Architecture as Code, which are essential components of DevOps. We combine engineering activities utilizing distributed version control and the W3C Web of Things to bring the associated advantages of pure software applications to an industrial CPS [5].

### 1.1. Contribution to the Work

- An increasing number of businesses and industries are turning to industrial automation to take advantage of cutting-edge technologies and improve productivity. Consequently, we created an ESSANN algorithm to advance the automated manufacturing sector.
- Industrial data comprising the cultural factors of elevator HPUs were made available for this study.
- Data collection is preceded by transformation, extraction, and management.
- The principal component analysis method was used in the process of feature extraction, and the least absolute shrinkage and selection operator methods were utilized in the process of selecting the most relevant features.
- As part of the evaluation process for the proposed work, metrics such as accuracy, precision, recall, MAE, delay, network capacity, scalability, computation time, packet loss, and operational cost were compared with those of previously developed approaches.

### 1.2. Motivation

In industrial automation operations, robots or computer-based control systems are used to monitor and manage machines and processes. Automation has the potential to boost dependability and output in an industrial context, while minimizing waste and errors, increasing security, and enhancing production process flexibility. Ultimately, industrial automation increases safety, reliability, and output. Using these technologies, businesses and people can efficiently satisfy their commercial and non-commercial demands. Organizations are compelled to automate industrial activities because of the high-risk administration and inefficiency of conventional techniques. This motivated us to develop the ESSANN algorithm to significantly improve the automation industry’s capacity to govern and monitor the structure of the industry without incurring delays or security problems.

### 1.3. Problem Statement

In our modern age, everything is required to be automated. Until recently, cameras were the only tools available to monitor conditions. We have deployed IoT in industry to monitor and alert authorized employees to take relevant actions in an attempt to eliminate human overhead; however, this will only partly satisfy our demand. Because this procedure may sometimes run overdue and cause damage to both businesses and people, it also improves the security and flexibility of the manufacturing system while reducing waste and increasing dependability and productivity. To achieve this goal, we are working on establishing a system for industrial automation that uses machine learning and is enabled by a smart industrial automation system built on the support of the Internet of Things.

### 1.4. Organization of the Research

Section 2 provides an overview of the related work. In Section 3, the implementation analysis is discussed, and in Section 4, the proposed solution architecture is explained. The performance analysis is presented in Section 5. Finally, the proposed method is presented in Section 6.

## 2. Related Works

Newer versions of many programs incorporate intelligent features that make using software more pleasurable by taking advantage of knowledge such as IoT and machine learning. The research in [6] presented the ‘Cluster-Tree-based Energy Efficient Data Gathering (CTEEDG)’ protocol to extend the lifespan and wireless sensor networks (WSN) efficiencies. CTEEDG employs fuzzy logic to identify the Cluster Head (CH), depending on the information gathered locally. The article [7] proposed the ‘Enhanced Principal Component Analysis and Hypergraph based Convolution Neural Network (EPCA-HG-CNN)’ technique, which is divided into two stages, such as enhanced PCA for feature reduction and a detailed and precise CNN for anomaly detection. The article [8] suggested a ‘Many-Objective Optimization Algorithm Based on the Dynamic Reward and Penalty mechanism (MaOEA-DRP)’ to enhance the verification significance of the block design for the improvement of public blockchain performance and reduction of malicious node gathering. The authors of [9] evaluated a sophisticated decentralized approach in which an ‘Automated Guided Vehicle (AGV) could optimally integrate charging stations into a tour of job locations. Modern industrial AGV systems employ algorithms to control the entire fleet of AGVs reliably and effectively. 

The research in [10] aimed to discover and research the potential for automation technologies enabled by 5G across a range of industries. They also examined the origins and advancements of pervasive computing devices, concentrated on how innovative 5G networks examined their critical empowering techniques, assessed their issues and challenges, explored their implementations in a range of industries, and highlighted how they will herald in a time of unrestricted communication, intelligent systems, and industry digitization. The article [11] provides “semi-supervised multi-scale convoluted feed-forward neural networks”. A one-dimensional multiscale convolutional layer was used as the generator, and an adverse approach was used to develop the model. Research in [12] demonstrated a ‘SCADA’ program written in the C# environment and extensively tested for use in controlling and monitoring industrial automation processes. SCADA software can be used to remotely monitor and control a facility’s data and log that data to an Internet of Things server. That article [13] stated that ‘edge computing’ has boosted real-time big business applications and advanced robotic processing of data. These cover edge computing and factory efficiency. The research in [14] organized the sensors and PLC to automate the bag-counting process. The project also intends to use SCADA for local control of the manufacturing process and monitoring, gathering, and processing real-time data. The authors of [15] developed a Chameleon Authentication Tree (CAT), a unique basic mechanism that increases efficiency and enables trustworthy information domain inquiries that can be confirmed. 

The work in [16] illustrated the obstacles faced by automation in an industrial environment and how cloud computing might be used to mitigate these concerns. In industry, automated methods boost output and improve accuracy. Productivity and precision greatly increase retailers’ earnings. The article [17] described the outcomes of inverse kinematic analysis for two application scenarios: point-to-point cycloidal trajectories and oceanic wave motions. The real-time direct kinematic calculation efficiency was also investigated. In [18], a WSN with a non-hierarchical structure was established. “Clique clustering” is a new protocol that, unlike prior proposals, incorporates a fail-safe technique to handle node failure or removal, both of which are common in WSNs. The authors of [19] reviewed the ideas of transmission and continuous learning. This review identifies potential techniques for commercial machine learning that use both groups of techniques for industrial automation. Therefore, the distinction between continuous and transmitting learning does not improve its application in any sector. In [20], the author defines the limitations placed on quality attributes by industrial automation, outlines the difficulties encountered by industrial IoT, and explores the possibilities presented by the use of various technological solutions. In [21], the author conducted a systematic literature review on the Industrial Internet of Things (I-IoT). The author introduced the I-IoT and described its architecture, applications (including manufacturing and process automation), and key features. The state of the art in research across the core facets of the system: control, networking, and computing is then considered. The author also classifies different types of industrial control systems and discusses some of the most current and important studies in this area. The work in [22,23] covered Industrial Automation utilizing the IoT. Advanced Industries ushered in a new era of physical manufacturing powered by an information economy. Industry 4.0 is the fourth industrial paradigm change, in which intelligent manufacturing technology is integrated with physical machinery. I-IoT combines industrial systems with powerful, near-real-time computing and analytics, enabled by low-cost, low-power sensor devices with global internet access. Table 1 indicates the summary of existing works.

## 3. Implementation Analysis

In addition to saving a significant amount of time and effort, automation enables a task to be completed very precisely. Robotic, command, and control systems and desktops are used in industrial automation to manage and perform specific processes. Therefore, we present the ESSANN method to considerably enhance the automation industry’s ability to regulate and analyze the industrial environment without experiencing any latencies or security flaws.

### 3.1. Analysis of the Dataset

An industrial dataset was provided by KLEEMANN Greece, including the cultural parameters of the elevator HPU which was made available. These measurements correspond to quality tests that monitor the speed of the elevator, pressure developed in the hydraulic unit, and noise produced during operation. The dataset includes 7200 unique examples that represent diverse customer orders and use different settings and specifications. An analysis of the presented dataset showed that the translational direction had no impact on the obtained slopes. As a result, orientation was not considered as a classification criterion, and the computed slopes for all orientations were combined into a single dataset of 2 × 7200 = 14,400 samples. However, there have been inconsistencies in the testing settings, such as some tests lasting longer than others or the HPU operating at a varied range of speeds. Such variances are inevitable because of the unique customer needs for every order; hence, data preprocessing is required, as shown in the next subsection.

The number of time steps captured in each HPU ranged from 201 to 1035; however, most samples (approximately 80%) only had 201 time steps. To make mini-batch processing possible, all data sequences were of the same length, which was 201 time steps. All speeds were within the same range, from 0 to 0.91. The noise and pressure had very different ranges: [0, 91.2] and [0, 53.98], respectively. To make them comparable, they were divided according to their highest values. The new dataset was divided into two parts: 90% for training and 10% for testing. (Sample time step variables) was the size of each dataset. Table 2 presents the sample datasets used.

### 3.2. Data Preprocessing

Data preprocessing refers to any operation carried out on the original data to prepare it for further analysis. Data pre-processing should combine disparate data sources with varying logic and phrasing to provide a unified computer source of information. Therefore, many operations must be executed, including data transformation, extraction, and management. In this section, we provide a solution in the form of a framework, complete with preparatory processes. The obtained data are used by the framework to create a unified representation of the data, and the model’s accessibility to the ML system paves the way for expert learning. Subsequently, the information was collected and entered into a dataset for further pre-processing. The three stages of preprocessing are data transformation, data extraction, and data management, which are used to verify and integrate the data. 

The mechanism of changing data from one place to another, usually from that of a source system to that required by a destination system, is known as data transformation. Data transformation was performed to examine the quality and feasibility of the data. The process of gathering or extracting various forms of information from numerous sources, many of which may be erratically arranged or entirely unorganized, is known as data extraction. Data extraction refers to the process of employing advanced methods to separate data from a system. Data management is the process of gathering, storing, and utilizing data in an expensive, accurate, and safe manner. Data control for several scenarios was made possible using a data management platform. Customer information can be gathered from many resources, analyzed, and organized using a data management platform.

### 3.3. Feature Extraction Using Principal Component Analysis (PCA)

PCA is a popular approach for analyzing massive amounts of data with many variables and attributes per inference. It is a quantitative strategy for lowering the dataset’s complexity to preserve the most material while improving comprehensibility. The algorithm is depicted below (Algorithm 1).
**Algorithm 1: PCA Algorithm**1. Determine the average attribute vector   $\alpha =\frac{1}{x}\;\sum_{n=1}^{x}G_{n}$ where *G_n_* is the design (*n* = 1 to *x*), *x* is the set of entries, and *x* is the attribute vector.
2. Calculate the correlation matrix
   $B=\frac{1}{x}$$\sum_{n=1}^{x}\left\{G_{n}-\alpha \right\}{\left\{G_{n}-\;\alpha \right\}}^{T}$ where *T* represents the transpose of matrix.

3. Compute Eigenvalue *τ_j_* and Eigenvector *u_j_* of the correlation matrix   $CV_{j}=\tau_{j}\;u_{j}$ (*j* = 1, 2, 3 … *m*), *m* = number of attributes

4. Estimate the high value of an Eigenvector.

(i)   Set up all of the Eigenvalues (*τ_j_*) in higher to lower order;

(ii)  Select the limit value, *θ*;

(iii) Number of high-valued *τ_j_* may be selected to fulfill the requirement   $\left(\sum_{j=k}^{T}\tau_{j}\right){\left(\sum_{j=1}^{y}\tau_{j}\right)}^{-1}$ ≥ *θ*, where *T* = number of high-valued *τ_j_* chosen;

(iv) Select Eigenvectors corresponding to selected high-valued *τ_j_*.

5. From the original attribute representation, retrieve the small attribute vectors (PCA).   *P* = *u^T^ G*, where *u* is the matrix of principal components and *G* is the matrix of the attribute. 

Generating the Eigenvalues of the covariance matrix derived from the feature space is a fundamental component of the PCA technique. Only a small number of characteristics, which correspond to those with strong Eigenvalues, will have discernible isolation from one another and may be considered for further analysis. The remaining features were disregarded. As a result, the complexity of the feature matrix is drastically reduced. 

Other names for Fisher discriminant analysis (FDA) include Fisher’s criteria, which is an extension of PCA. To calculate the discriminant distance $q_{n}^{x,y}$ between two classes, *x* and *y*, the mean and standard deviations of the selected features must be known. Equation (1) provides a formula for calculating the discriminant distance according to PCA.

(1) $$q_{n}^{x,y}=\frac{{\left|\left(Avg\;p_{n}^{x}\right)-Avg\left(p_{n}^{y}\right)\right|}^{2}}{{\left[Sv\left(p_{n}^{x}\right)\right]}^{2}+{\left[Sv\left(p_{n}^{y}\right)\right]}^{2}}$$

where the *n*th feature for the carrying circumstances *x* and *y*, correspondingly, is $p_{n}^{x}$ and $p_{n}^{y}$. The Fisher discriminant distance between the two categories of orientations *x*, and *y*, for the nth functionality, is 〖*q*〗_*n*^(*x*, *y*); Average () and Sample variance () are the average values and sample variations. It is evident that its dispersion inside categories is identical to the sum of their variances, which is the denominator of the above equation, and that the dispersion among categories is identical to the squares of the distance between their averages.

It depicts the visual distinction between two sets of data and, therefore, is nondimensional. Equation (2) contains the information for multiple (categories of both *x* and *y*) challenges. The discrepancy between the average scores and sample variations is well described.

(2) $$T_{n}^{x,y}=\frac{{\left|\left(Mean\;p_{n}^{x}\right)-Mean\left(p_{n}^{y}\right)\right|}^{2}}{{\left[Std\left(p_{n}^{x}\right)\right]}^{2}\;+{\left[Std\left(p_{n}^{y}\right)\right]}^{2}}$$

where the carrying requirements *x* and *y* belong to the nth features and functionality, and *n* is the separation index for that extracted features.

Construct each part of the attribute data for the extraction as an A-shaped vector.

(3) $$Y=\left[Y_{1},Y_{2},\ldots \ldots ,Y_{n}\right]$$

The sample has r points, where *n* is the total number of samples. The correlation matrix *S* is then computed as shown in Equation (4).

(4) $$T=\frac{1}{m}\sum_{k=1}^{m}\left(Y_{k}-\bar{Y}\right){\left(Y_{K}-\bar{Y}\right)}^{S}$$

(5) $$\mathrm{s.t.}\bar{X}=\frac{1}{n\;}\sum_{k=1}^{n}X_{k}$$

The example numerical value is *Y*. The Eigenvector *S* = [u1, u2,……, un] should be produced as *S* is an r r matrix, and its Eigenvalues are [$\delta_{1}$, $\delta_{2}$,……, $\delta_{n}$] ($\delta_{1}\geq \delta_{2}\geq$……$\delta_{2}\geq$). This Eigenvector serves as the orthogonal foundation for the data from the industry. A higher feature value may contribute more. Using normalized techniques to calculate the proportion, the proportion *Qk* might be calculated as shown in Equation (6).

(6) $$QK=\lambda k{\left(\sum_{i=1}^{m}\delta j\right)}^{-1}$$

Any Eigenvectors with a negligible feature value need to be disregarded. The model is restored by utilizing the initial *d* vectors of the restoration vector *Y*, where *Q* is the model. The correlation matrix is calculated to determine the components with the largest correlation, and these components are given much greater weight on the electro-attribute data for automation than the ones with the smallest correlation. This offers a means of achieving dimensionality reduction, considerably speeding up the retrieval of ideal features.

(7) $$X=\sum_{i=1}^{d}u_{i}^{T}Xu_{i}$$

### 3.4. Feature Selection Using Least Absolute Shrinkage and Selection Operator (LASSO)

The LASSO approach was employed to select the parameters and decrease the differences in the estimation. When there are many factors but few data in a sample, the regression shrinkage approach is widely used. This method reduces the residue sum of the squares under the condition that the total actual value of the factors is smaller than a constant, which is equivalent to reducing the sum of squares with such constant $\sum_{j}\left|\beta_{j}\right|\;\leq P$, and causes certain of the factors to be decreased to zero. By applying a penalty, the technique optimizes the target while using L1 regularization. The overall actual value of the factors makes up this penalty, which determines which factors and how much of them reduce. The LASSO estimate comes from

(8) $$\delta_{Lasso\;}=\frac{mininum}{\delta }\left(\sum_{a=1}^{k}{\left(u_{i}-\sum_{q}\delta_{q}V_{ij}\right)}^{2}+\Gamma \sum_{q}\left|\delta_{q}\right|\right)$$

where the shrinking factor is denoted by Γ.

LASSO is insufficient to fulfill the oracle attribute. Consequently, each coefficient has a weight function, which is specified in Equation (4).

(9) $$\delta_{Modified\;LASSO\;}=\frac{minimum}{\delta }\left(\sum_{a=1}^{k}{\left(u_{i}-\sum_{q}\delta_{q}V_{ij}\right)}^{2}+\;\Gamma \sum_{q=1}^{m}{\hat{W}}_{f}\;\left|\delta_{q}\right|\right)$$

W*_f_* is a weight function that is computed by $W_{f}=\frac{1}{{\left|{\hat{\delta }}_{f}\right|}^{\alpha }}$ where α is a positive factor, $\left|{\hat{and\;\delta }}_{f}\right|$ is the initial computation of the δ factor. 

The LASSO algorithm is described below (Algorithm 2).
**Algorithm 2: LASSO Algorithm**Start *B* = *P* + *δJ*; the Magnitude of *B* does not vary.Repeat for *q* = 1, 2 … *m*, and so on until convergence:
(a)Divide matrix *B* into Section 1: all but the *q*th row and column, and Section 2: the *q*th row and column.(b)Compute the solution for the estimating equations *B*_11_*γ* − *P*_12_ + *δ*. Assign (*γ*) = 0 utilizing the cyclical coordinate-descent algorithm (17.26) for the advanced LASSO.(c)Increase $b_{12}=B_{11}\hat{\delta \;}$.In the last step (for each *q*), solve for ${\hat{\vartheta }}_{22}=-\hat{\delta }$ $.{\hat{\vartheta }}_{22}$, with $\frac{1}{\hat{\vartheta_{22}}}=b_{12}{}^{T}\hat{\delta }.$

## 4. Proposed Solution

### 4.1. Elaborative Stepwise Stacked Artificial Neural Networks (ESSANN)

**Figure 2:**
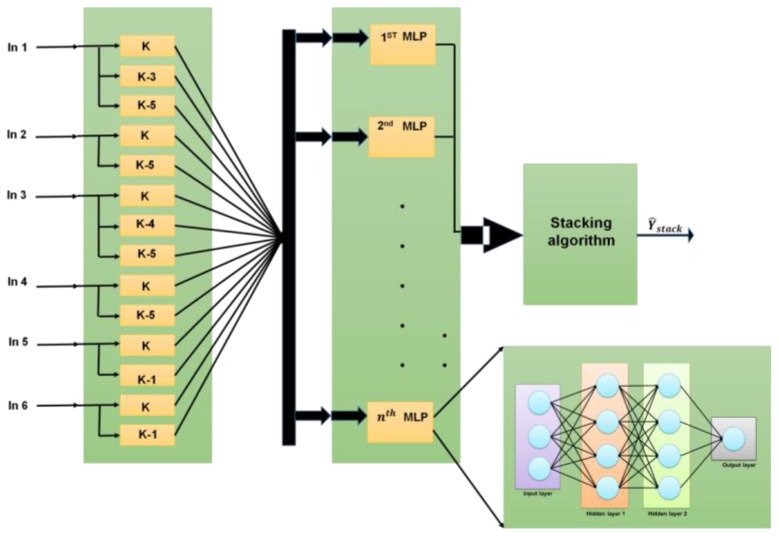
Elaborative stepwise stacked artificial neural networks. Level of input: It receives inputs given by the user in a variety of ways. Level of hidden: Hidden levels are shown between the input and output levels. It performs all the computations necessary to uncover the connections and hidden characteristics. Level of output: This layer is used to communicate the output after the data have undergone several alterations in the hidden layer.

In a very short period, artificial neural networks can analyze massive amounts of data with complicated characteristics and isolate various patterns. Consequently, they are helpful for a wide range of commercial functions, including industrial automation, spotting data abnormalities or mistakes, and picking up certain sights, noises, or visuals. With limitless supplied inputs, they may employ identity to deliver the best results. A collection with the ESSANN for all conceivable variables measured was proposed to provide sufficient guidance. First, in a StackWise neural network, particular combination strategies of MLPs based on a simple average, the least-squares approach, and a nonlinear combination strategy based on a cascade of neural networks were considered. The performance of the different models was compared using the entire set of patterns. A schematic of the proposed structure is shown in Figure 2. The stacking strategy was implemented on a subset of the available suboptimal models after they were sorted based on their modeling performance. Thus, better-working MLPs were considered first. The input fed to the proposed ESSANN model was partitioned into subsets. In Figure 2, *k* indicates *k*th instance to be analyzed by the model. ESSANN model is a group of ‘*n*’ MultiLayer Perceptron (MLP) models stacked over each other. Each MLP layer consists of an input layer, multiple hidden layers, and an output layer. The process involved in each MLP layer is explained below. The industrial data to be analyzed is provided as input to the first MLP layer. The first MLP layer processes and analyzes the industrial data to output the industrial decision. The output of the first MLP layer is sent as input to the second MLP layer. In this way, data are propagated from the previous MLP layer to the next MLP layer in the ESSANN model. As the data reaches the *n*th MLP layer, the output of the *n*th MLP layer is presented as the final output of the ESSANN model.

In an MLP neural network, it is particularly important to choose the number of hidden layers and the number of neurons in each layer. In a small dataset, too many hidden layers will not only make the model more complicated, but also lead to overfitting of the model and poor model generalization ability. Therefore, in small datasets, one or two hidden layers of MLP neural networks are generally used for modeling. We established one hidden layer and two hidden layers of MLP models and chose the model with the least error as the final prediction model of the pollutants. To solve this issue, a stepwise model was established through the influence of various factors on industrial automation systems, and it was used to provide the fitted value of the automation system at the corresponding moment. Then, the stepwise MLP neural network model was established by taking the fitted value and other data and time measured by the self-built point as input values and the national control point data as output values. An algorithm was employed to determine the elaborative stepwise stacked artificial neural network parameters for each pairing. The algorithm for ESSANN is depicted below (Algorithm 3).
**Algorithm 3: ESSANN**1: **ESSANN approach** (Input, Neurons, Iteration)   Generate a source database

2:  Input
← a database that includes every variation of variables that might exist Equip ESSANNs

3:  **for** Input = 1 to *n*
**do**

4:        **for** Neurons = 1 to *n*
**do**

5:    **for** iterate = 1 to *n*
**do**

6:        Equip ESSANN

7:        ESSANN-Storage
← saves the highest value

8:    **end for**

9:  **end for**

10:   ESSANN-store
← based on inputs, preserve the most accurate forecasting ESSANN

11:   **end for**

12: **Return ESSANN-Storage** ESSANN for all variable combination

13: **end approach**

ESSANNs are non-parametric machine learning techniques that comprise a matrix of linked neurons. Based on an input signal, the neuron weights and the inputs are given. In each MLP layer, the input layer has data, random weights, and bias term. These then pass through the hidden layer, and it then outputs the result. After this, the model learning error is determined, and finally, based on error, the model weights are updated. This weight updating is done continuously until we get a satisfactory error rate. This iterative weight updating process is applied to each MLP layer in the ESSANN model. The generation of the output signal depends on the signal. This signal is then sent and may activate more neurons depending on the structure of the network. In contrast, machine learning (ML) is related to the learning representation of data. The ESSANN, as these are often called, is motivated by the organization and function of the brain. The method uses a pyramid of ideas in a subject area to help the machine learn from experience. Because this information is obtained automatically, this method does not require manual interaction to offer machine expertise. A pyramid of ideas makes it easier to divide complicated ideas into several levels of easier things. When there are several processing levels, ML approaches learn through several different layers. This method has been used in industries such as robotics, genetics, and pharmaceuticals. ESSANN in industrial automation contributes to increased efficiency, reliability, and efficiency, all of which reduce operating costs. However, a decrease in production costs is the main benefit of an industrial automation system. This can be achieved by employing the proposed method. We must first understand the structure of a neural network to understand the structure of an elaborative stepwise stacked artificial neural network. A massive amount of elaborate artificial neurons, also known as modules, are assembled in a hierarchy of levels to construct a neural network. 

### 4.2. Benefits of ESSANN

- Flexibility in handling several tasks concurrently. The ESSANN has a specific value that is capable of doing so.
- The material used in conventional computing is saved all over the system, not just in a database, as opposed to the database itself. The system continues to function, even if some material temporarily disappears from one location.
- Considering storage allocation: For ESSANN to be capable of adapting, it is crucial to identify the instances and to motivate the system by presenting such instances to the system to produce the desired output. The system outcome may be incorrect if the action cannot be represented by the system in all of its characteristics, since the system’s continuity is approximately equal to the selected occurrences.

### 4.3. Drawbacks of ESSANN

- Guarantee of an appropriate network framework. There is no set formula for determining the structure of an ESSANN. The correct network model is achieved through practice, effort, and failure.
- Unknown networking attitude. This is ESSANN’s most important problem. The ESSANN does not offer explanations about why or how it came up with a test solution. This erodes the confidence of the system.
- Technology constraint: Owing to the nature of ESSANN, simultaneous processors are required. Consequently, the realization of the equipment is dependent.
- Statistical data may be used using ESSANN. Before issues are presented to ESSANN, they must be transformed into numbers. The platform’s efficiency is significantly affected by the presentation technique, which must be determined here. This depends on the user’s skills.
- The system is restricted to a certain standard error, and this relative error does not provide the best outcomes.

## 5. Performance Analysis

Integrating the IoT and machine learning has potential in the contemporary age of automation and smart computing. We proposed the elaborative stepwise stacked artificial neural network (ESSANN) technique to create a system that can autonomously monitor applications in the industry, produce notifications or warnings, or make intelligent decisions utilizing the IoT idea. The parameters used to compare the proposed method to existing methods include delay, network bandwidth, scalability, computation time, packet loss, and operational cost. The efficacy of the proposed approach is compared to existing techniques, such as the Unified Artificial Immune System (UAIS), 5G-enabled IoT and Blockchain (Blockchain 5G-IoT), Blockchain-based Cyber Threat Mitigation System (BCMS), and clock synchronization techniques (CST).

### 5.1. Delay

**Figure 3:**
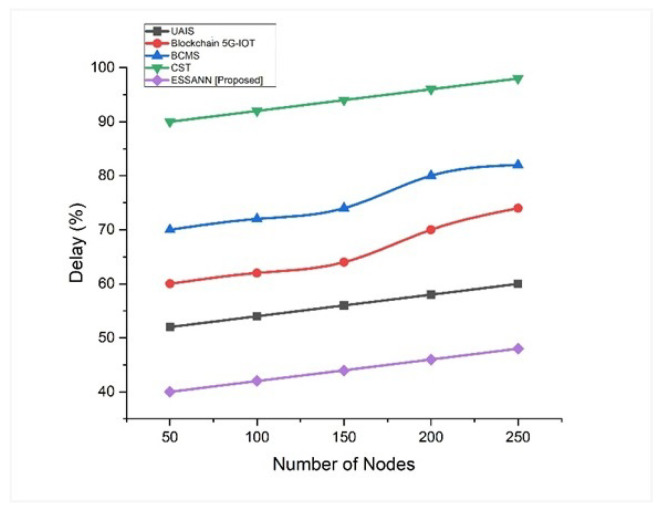
Comparison of delay [23,24,25,26].

The delayed state is the instant beginning of the process of delaying, blocking, or pushing objects to move more slowly than normal. Delay for automated decision-making process in industries is calculated by Equation (10). Their current automated decision-making process is slow. When the results of the experiment were compared with those of the previously known technique, as shown in Figure 3, we discovered that a low-level delay was obtained using the ESSANN methodology. ESSANN shows a 52% delay for 250 nodes. This proves that delay regarding the industrial automated decision-making process based on ESSANN was minimal compared to existing industrial automation techniques like UAIS, BCMS, CST, and Blockchain 5G-IoT.

(10) $$y\left[n\right]=x\left[n-n_{0}\right]$$

### 5.2. Network Bandwidth

**Figure 4:**
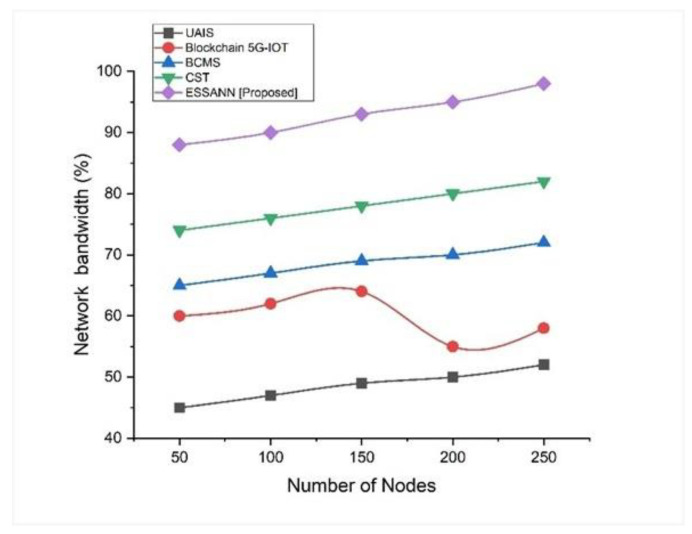
Comparison of network bandwidth [23,24,25,26].

Network bandwidth is a measurement of a network’s capability or information transmission rate. Bandwidth required for automated data transmission in industries is calculated by Equation (11). This is an essential connection characteristic for comprehending a network’s performance and reliability. This demonstrates how the proposed method automates data transmission in the industry. Figure 4 shows the network bandwidth. The ESSANN obtained 97% bandwidth for 250 nodes, which was higher than that that obtained by existing approaches. Therefore, the proposed approach is more efficient than existing systems. Higher network bandwidth achieved by industrial automation systems relying on the proposed ESSANN model ensures the higher speed of industrial data transmission in industrial network.

(11) $$H\left(e^{j\hat{\omega }}\right)=e^{-j\hat{\omega }n_{0}},\left|H\left(e^{j\hat{\omega }}\right)\right|=1$$

### 5.3. Scalability

**Figure 5:**
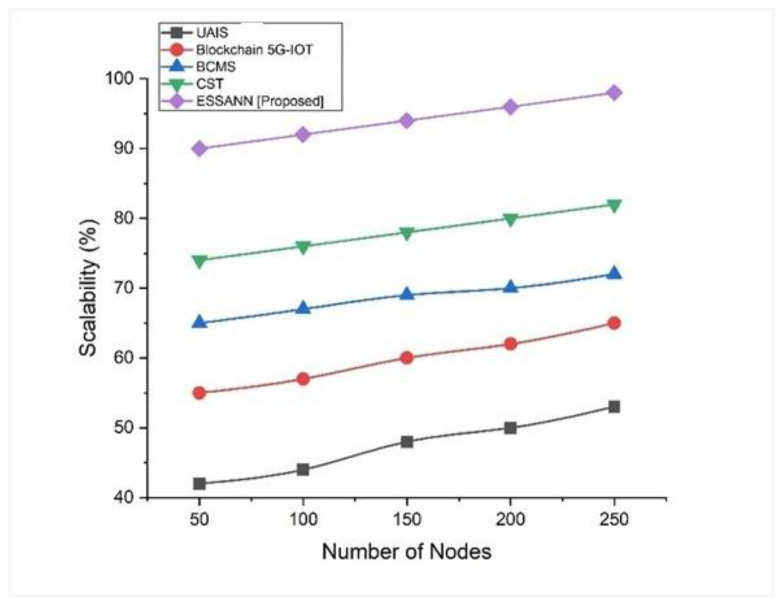
Comparison of scalability [23,24,25,26].

The ability of a system to adapt its performance and efficiency to changing applications and industrial automation needs is referred to as scalability. Based on the ESSANN methodology, we found that the scalability was 96% for 250 nodes, as depicted in Figure 5. Scalability observed for the ESSANN which was suggested for industrial automation system was higher than that of existing industrial automation techniques like UAIS, BCMS, CST, and Blockchain 5G-IoT. This shows that the industrial automation process can be effectively enhanced by utilizing the proposed ESSANN model in industrial networks.

(12) $$\alpha_{j}\left(s,t\right)=\frac{1}{st}log\;log\;E\left[e^{2X,\left|0,r\right|}\right]\;$$

### 5.4. Computation Time

**Figure 6:**
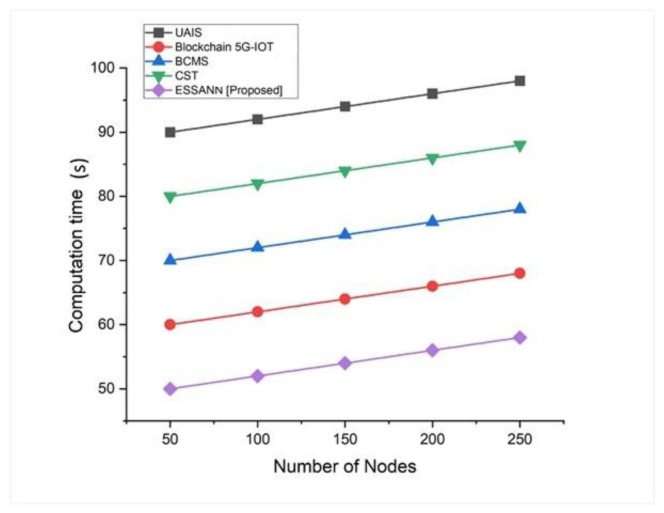
Comparison of computation time [23,24,25,26].

Computation time is the length of time required to accomplish a calculation (sometimes known as “running time”). Computation time for automated decision making was calculated by Equation (13). It shows how promptly our model detects the problem and makes a decision. Figure 6 shows the computation time. ESSANN obtained 59 s for 250 nodes. Therefore, the proposed approach takes less time than the existing systems. These findings suggest that utilizing ESSANN in the industrial automation process helps in reducing the computation time for industrial problem detection and decision making.

(13) $$C_{Time}=C-Clock_{Cycles}*Clock\_Cycle_{Time}\;=\frac{C\_Clock\_Cycles}{Clock\_Rate}$$

### 5.5. Packet Loss

**Figure 7:**
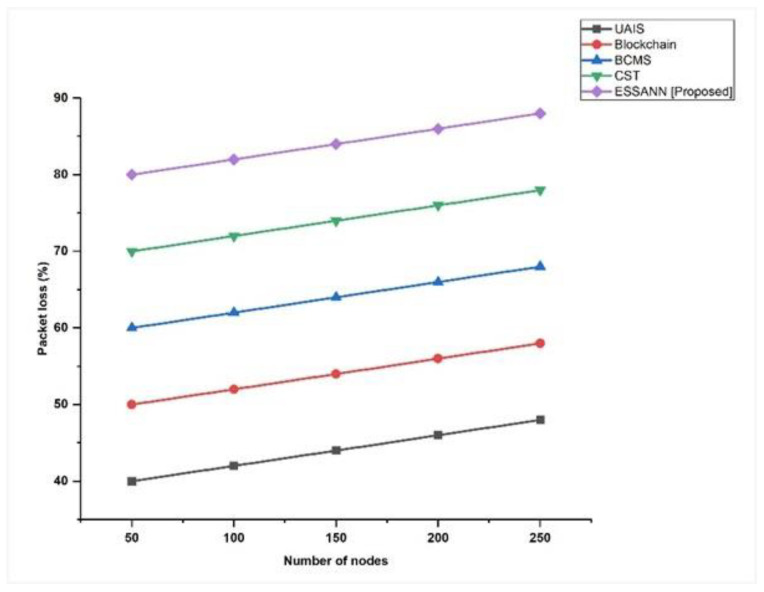
Comparison of Packet loss [23,24,25,26].

Packet loss occurs when a small number of supplied data packets are misplaced inside a packet. Consequently, there may be performance issues in all types of industrial automation. Packet loss involved in the industrial network was calculated using Equation (14). Figure 7 compares the experimental results with those of the previously recognized methodology, and we discovered that the decreased packet loss had scores of 53% for 250 nodes based on the ESSANN technique. It was confirmed that the number of industrial data packets lost during transmission can be effectively minimized by the proposed ESSANN model in industrial automation systems.

(14) $$Packet\;Loss=\frac{\left(Total\;Tx-Total\;Rx\right)}{Total\;Tx}\times 100\%$$

### 5.6. Operational Cost

**Figure 8:**
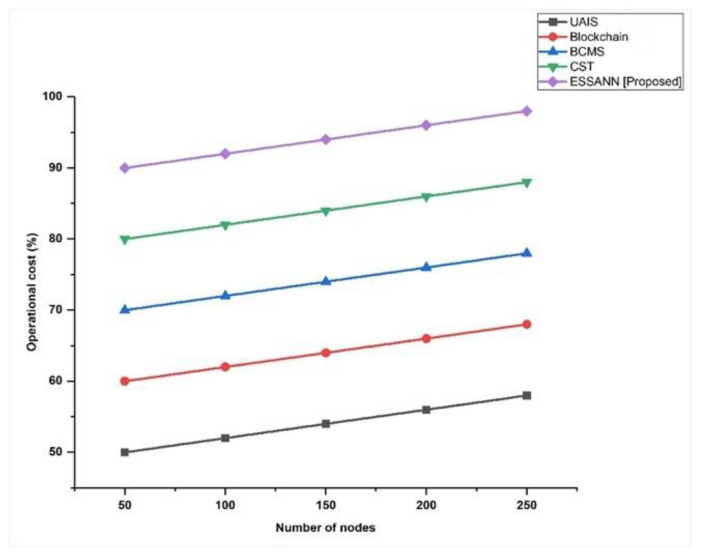
Comparison of operational cost [23,24,25,26].

The continuous expenditures for deploying industrial automation utilizing our strategy are known as the operational costs. Figure 8 shows the operational costs. The ESSANN achieved 59% accuracy for 250 nodes. Therefore, the proposed approach is less costly than existing industrial automation techniques like UAIS, BCMS, CST, and Blockchain 5G-IoT. The results observed from Figure 8 illustrated that deploying industrial automation utilizing ESSANN strategy is cost-effective.

(15) $$Operating\;leverage=\frac{Fixed\;costs}{\;total\;Costs}$$

### 5.7. Accuracy, Precision, and Recall

**Figure 9:**
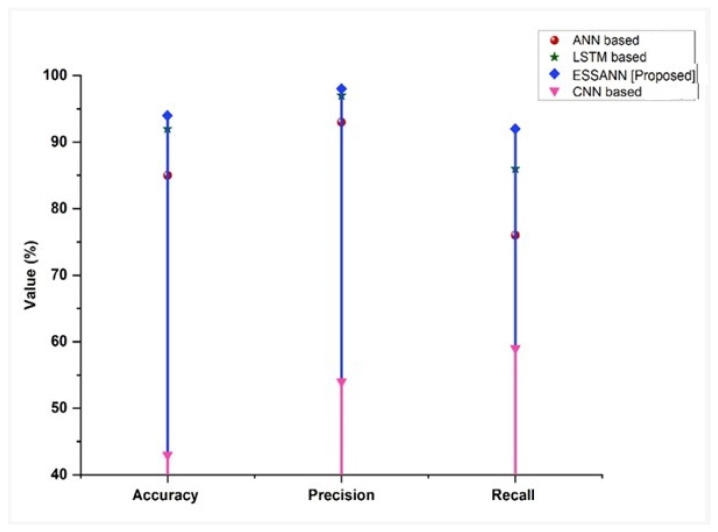
Comparison of accuracy, precision, and recall.

In industrial automation, two measurements of observational error are known: accuracy and precision. Accuracy refers to how close a certain collection of measures is to their actual value, whereas precision refers to how close the measurements are to one another. Recall, which is synonymous with sensitivity, refers to the percentage of total significant samples collected. Figure 9 shows the accuracy, precision, and recall comparisons of the proposed ESSANN with those of existing methodologies. From the figure, it is clear that the proposed approach has a higher accuracy, precision, and recall percentage. The results observed in this study depicted that application of ESSANN in the decision-making process was highly efficient for industrial automations.

### 5.8. Mean Absolute Error

**Figure 10:**
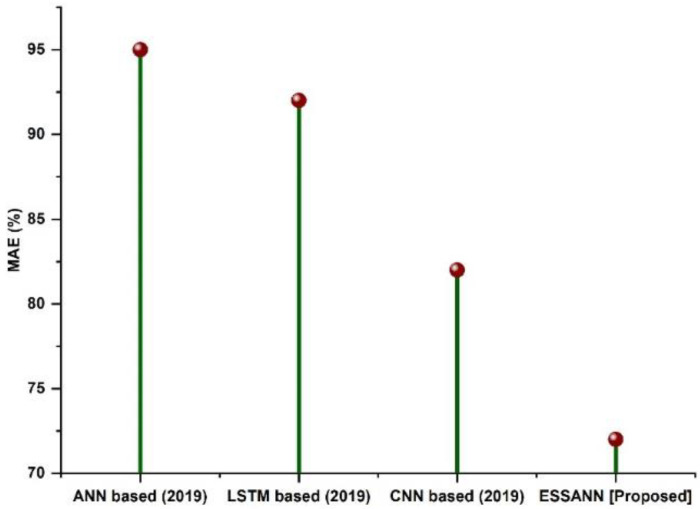
Comparison of MAE.

In industrial automation, the difference between the measured or inferred value of a quantity and its actual value is known as the mean absolute error. Figure 10 shows the MAE comparisons of the proposed ESSANN with the existing methodologies. From the figure, it is clear that the proposed approach has less MAE percentage when compared to existing methodologies. Error in industrial process-related decision making using ESSANN was very much reduced compared to conventional models. This in turn indicates that employing ESSANN in industrial automation system results in appropriate decisions regarding industrial problems.

A comparison of the recommended technique with the existing models is shown in Figure 3, Figure 4, Figure 5, Figure 6, Figure 7, Figure 8, Figure 9 and Figure 10. Existing methodologies such as UAIS, Blockchain 5G-IOT, BCMS, and CST were used in this investigation. Owing to the limitations of the already-used methods, the proposed strategy is superior to the existing methods in terms of its performance. The disadvantages of the approaches already in use are as follows. Modern oil refinery processes involve large amounts of material and rapid assessment of performance data, which raises the chance of making poor selections regarding the management of sophisticated high-tech machinery. As a result, [24] suggested the creation of a unified artificial immune system (UAIS) as an advanced system for the management of complicated assets in the oil and gas business. It has a very low reliability. The authors of [25,26] suggested a 5G-enabled IoT as the foundation for blockchain (blockchain 5G-IOT)- based industrial automation for use in smart cities, digital homes, healthcare 4.0, intelligent farming, automated cars, and supply chain administration, among other industries. IoT-integrated industrial automation provides an effective decentralized access control method for device-to-device (D2D) connectivity in numerous industrial domains. The authors of [27,28] suggested a Blockchain-based Cyber threat Mitigation System (BCMS), which would identify barriers to industrial automation in businesses, provide a grid for intelligent vehicle communication, and provide techniques for putting together an automation process among connected vehicles. Data transactions occur at an extremely slow rate. The authors of [29,30] suggested employing clock synchronization techniques (CST) for further industrial automation, enhanced information conveyance, and the addressing of protection challenges. Its cost of computing is quite high.

## 6. Conclusions

Industrial automation has recently gained increasing support from a variety of sectors owing to its many advantages, including higher efficiency, reliability, and security at cheap prices. As a result, we may save time and make fewer mistakes compared to physically repeating repetitive instructions. In this paper, we proposed an elaborative stepwise stacked artificial neural network (ESSANN) algorithm to greatly improve automation industries in controlling and monitoring the industrial environment. The simulation results achieved better performance when compared to the existing methods in terms of delay time (52%), network bandwidth (97%), scalability (96%), computational time (59 s), packet loss (43%), operational cost (59%), accuracy (98%), precision (98.95%), recall (95.02%), and MAE (80%). The application of industrial automated systems by organizations improves safety, frees up energy, improves productivity, minimizes monitoring, and reduces expenses. These advantages help businesses operate more profitably, productively, and efficiently. Industrial automation cannot perform difficult or non-repetitive operations because of the efficiency and high availability gains provided by the machines. Consequently, complex production-related challenges remain unsolved using existing automation technologies. Hence, advanced optimization algorithms may suit the future scope of research.

## Figures and Tables

**Table 1:** The Summary of existing works.

Reference	Algorithm	Performance Metrics	Objective
**Karunanithy and Velusamy [6]**	Cluster-Tree-based Energy Efficient Data Gathering (CTEEDG)	Membership function for input and output, average energy consumption in joules, and average end-to-end delay in seconds are evaluated.	Integration of WSN and IoT
**Krithivasan et al. [7]**	Enhanced Principal Component Analysis and Hypergraph-based Convolution Neural Network (EPCA-HG-CNN)	Accuracy, mean square error, time efficiency, and runtime in seconds are used as performance metrics.	SCADA system
**Cai et al. [8]**	Many-Objective Optimization Algorithm Based on the Dynamic Reward and Penalty mechanism (MaOEA-DRP)	The average value in BFE functions.	Industrial Internet of Things (I-IoT)
**De et al. [9]**	Sophisticated Decentralized Approach	Resource level and total average time are used.	Automation
**Attaran [10]**	This article reviews how 5G can enable or streamline intelligent automation in several industries.	Average throughput, flexibility, delay, limited bandwidth, dependability, high accuracy, range, and energy consumption are some of these assessment criteria.	Evaluation of 5G
**Pan et al. [11]**	Semi-Supervised Convolutional Generative Adversarial Network	Classification accuracy and average accuracy.	Industrial automation system
**Phuyal et al. [12]**	Supervisory Control and Data Acquisition (SCADA)	DC bus voltage, output voltage, load current, frequency, and speed.	SCADA software
**Stankovski et al. [13]**	Edge computing	Comparison of traditional concepts on edge computing in industrial automation.	Big data
**Banaulikar et al. [14]**	Programmable Logic Controller (PLC) and SCADA	Speed, default bag length, and current bag length are used as performance metrics.	PLC
**Xu et al. [15]**	Chameleon Authentication Tree (CAT)	Time and average time are assessed.	Verifiable Data Streaming (VDS)
**Mentsiev et al. [16]**	Cloud computing	Enhance analysis of data and enhance consolidation of workload.	Cloud computing
**Silva et al. [17]**	Stewart Platform robot	Time evolution for the generation of Cartesian motion.	Motion control automation
**Saravanan and Solairaju [18]**	Wireless Sensor Network (WSN)	Backbone reorganization is evaluated.	WSN
**Maschler and Weyrich [19]**	Ideas of transmission and continuous learning	Analyzing anomalies, time series forecasts, visual computing, fault finding.	Deep learning

**Table 2:** Utilization of datasets.

Set of Data	Sample Size
Training samples	13.968 × 312 × 5
Testing samples: A	1.625 × 312 × 5
Testing sample: B	1.625 × 312 × 5

## Data Availability

There are no available data to be stated.
